# Ghrelin and LEAP2: Their Interaction Effect on Appetite Regulation and the Alterations in Their Levels Following Bariatric Surgery

**DOI:** 10.3390/medicina61081452

**Published:** 2025-08-12

**Authors:** Nese Alic, Aylin Ayaz

**Affiliations:** 1 Department of Nutrition and Dietetics, Faculty of Health Sciences, Munzur University, 62000 Tunceli, Turkey; 2 Department of Nutrition and Dietetics, Faculty of Health Sciences, Hacettepe University, 06100 Ankara, Turkey; baylin@hacettepe.edu.tr

**Keywords:** ghrelin, LEAP2, acyl ghrelin, des-acyl ghrelin, appetite regulation, bariatric surgery, sleeve gastrectomy, Roux-en-Y gastric bypass, obesity

## Abstract

*Background and Objectives*: Ghrelin plays key roles in appetite regulation, involving both homeostatic and hedonic pathways. In obesity, its metabolism is affected by alterations in neural and hormonal responses. Liver-Enriched Antimicrobial Peptide 2 (LEAP2), recently identified as an endogenous ghrelin receptor antagonist, has emerged as a potential regulator of appetite and energy balance, and bariatric surgery is known to induce changes in this system. In this review, we aimed to evaluate the roles of acyl ghrelin, des-acyl ghrelin, and LEAP2 in food intake regulation and summarize findings on the alterations in their levels after bariatric surgery. *Materials and Methods*: We conducted a narrative review of human and animal studies published in the literature investigating the roles of acyl ghrelin, des-acyl ghrelin, and LEAP2 in food intake regulation, as well as changes in their levels in obesity and following bariatric surgery. *Results*: Alterations in the ghrelin system, i.e., the acyl ghrelin and des-acyl ghrelin isoforms and LEAP2, in obesity have been reported. Experimental studies have shown that LEAP2 administration reduces food intake and body weight by suppressing ghrelin-induced food intake. Most studies have found marked reductions in fasting acyl ghrelin after sleeve gastrectomy, more so than after Roux-en-Y gastric bypass. *Conclusions*: In obesity, altered neural and hormonal responses to food also affect ghrelin metabolism, with significant deviations in acyl ghrelin levels and impaired appetite regulation mechanisms. Unlike ghrelin, LEAP2 levels tend to be elevated in obesity. While changes in acyl ghrelin and des-acyl ghrelin isoforms, particularly after sleeve gastrectomy, are well documented, data on LEAP2 remain limited. Further research is needed to better define the role of LEAP2 in ghrelin-mediated food intake and its potential as a therapeutic target in obesity.

## 1. Introduction

According to the World Health Organization (WHO), obesity is a chronic disease characterized by excessive body fat and is associated with physical, mental, and social problems [1]. It involves abnormalities in energy metabolism, appetite regulation, and the endocrine system [2]. Nutrition is essential to the survival of all living beings, as it provides substrates for energy metabolism regulated by the brain’s hedonic and homeostatic systems [3]. However, excess nutrient intake can act as a metabolic toxin in cells [4]. Therefore, global strategies are being developed to prevent overeating and combat obesity [5], and understanding hunger and satiety mechanisms has become increasingly important in addressing the obesity epidemic [6].

Energy homeostasis is primarily regulated by neuronal circuits in the hypothalamus and brainstem, while reward and motivation in eating behavior are mediated by neurons in limbic regions and the cerebral cortex [7]. Both homeostatic and non-homeostatic (hedonic) mechanisms integrate metabolic signals and cognitive processes to regulate food intake [8,9].

Ghrelin, mainly secreted by the stomach, plays a key role in energy homeostasis by stimulating growth hormone release, increasing appetite, and promoting weight gain [10,11,12]. Studies suggest that in obese individuals, ghrelin levels are markedly lower than in normal-weight individuals and appetite regulation mechanisms and ghrelin transport across the blood–brain barrier are impaired [13,14,15]. These findings support the hypothesis that the hypothalamic system may develop resistance against the effects of ghrelin in the obese population [15].

Since the discovery of ghrelin, the mechanisms regulating its secretion and controlling its effects have not been clearly defined. However, progress in ghrelin regulation research was made in 2018, when Liver-Enriched Antimicrobial Peptide 2 (LEAP2) was identified as the first endogenous antagonist of the ghrelin receptor [16]. Subsequently, LEAP2 was reported to be an endogenous circulating hormone [17] and to block the main effects of ghrelin caused by its binding to GHS-R, including food intake, growth hormone (GH) release, and the maintenance of serum glucose levels during chronic energy restriction [16]. Comparisons of obese and normal-weight individuals show that LEAP2 levels tend to increase in obesity in contrast to ghrelin [14,15].

Current obesity treatments focus on lifestyle interventions, pharmacotherapy, and bariatric surgery. The latter induces anatomical and hormonal changes and is considered to be the most effective [18,19,20,21]. Bariatric surgery, especially sleeve gastrectomy (SG) and Roux-en-Y gastric bypass (RYGB), reduces hunger and alters food preferences, partly due to its effects on the gut–brain axis [22,23,24,25]. Ghrelin, a key gut–brain hormone, is linked to post-surgical weight loss [26]. Researchers have reported that plasma ghrelin levels decrease with the removal of the fundus region of the stomach, especially after SG [14,26]. It has also been reported that the decrease in plasma ghrelin levels in the period immediately after sleeve gastrectomy may induce weight loss by decreasing appetite, whereas a tendency for ghrelin levels to increase in the long term may negatively affect the long-term success of SG [27]. However, only total ghrelin hormone levels have been examined in the literature, representing a research gap. It is possible that the ratio between acyl ghrelin (AG) and des-acyl ghrelin (DAG) changes without changes in the concentration of total ghrelin in plasma [28].

The association of LEAP2 with ghrelin has been recently described, and changes in the levels of these hormones after bariatric surgery have already attracted scientific interest. However, there is a limited number of related studies. In this review, we aim to evaluate the roles of acyl ghrelin, des-acyl ghrelin, and LEAP2 in appetite regulation and the changes in their levels following bariatric surgery.

## 2. Appetite Regulation: Homeostatic and Hedonic Pathways

Nutrition provides substrates for energy metabolism and is regulated by the brain’s homeostatic and hedonic systems [3]. While adequate nutrition supports health, excessive intake leads to metabolic overload and related diseases, such as obesity [4,29].

Food intake and energy expenditure are key regulators of body weight. To control the former, the brain integrates both physiological and hedonic signals, continuously updating the body’s energy status and regulating hunger and satiety responses through peripheral signals and the neuronal pathways of the gut–brain axis [30]. Energy homeostasis is maintained by hypothalamic and brainstem circuits, while reward and motivation involve the limbic system and cortex [7]. Eating behavior is influenced not only by homeostatic needs but also by psychological, social, and cultural factors [9]. Food intake and its underlying biological parameters are, therefore, often divided into two main categories: (i) homeostatic processes based on energy and metabolic deficiencies and (ii) non-homeostatic (hedonic) processes driven by environmental/cognitive factors [8].

### 2.1. Homeostatic Control of Food Intake

Homeostatic hunger is the physiological desire to eat that occurs in response to energy deficit and aims to maintain energy homeostasis. The hypothalamus plays a key role in homeostatic control [31]. In 1940, Hetherington and Ranson demonstrated the importance of two regions of the hypothalamus—the ventromedial and lateral hypothalamic areas—in the regulation of eating behavior and the metabolism of hunger and satiety [32]. Similarly, in the mid-1900s, many other studies also identified the hypothalamus as the key brain region that regulates energy balance and controls food intake [33,34]. While satiety is regulated by the ventromedial nucleus, hunger is controlled by the lateral hypothalamic area. It has been reported that the appetite control mechanism involves a complex neural network, including different pathways within specific hypothalamic nuclei and various regulators [31]. Another component of homeostatic control, the arcuate nucleus (ARC) of the hypothalamus, plays a critical role in the regulation of food intake and metabolism [35]. The ARC is situated near the median eminence (ME), a circumventricular organ that is rich in fenestrated capillaries and lacks a blood–brain barrier. This anatomical position allows the ME to facilitate the transport of peripheral hormonal and nutrient signals to ARC neurons. In turn, the ARC integrates these metabolic and hormonal signals from the peripheral circulation with both peripheral and central neural inputs, forming a coordinated feedback mechanism [36]. The peripheral signals received by the ARC affect two distinct neuronal subtypes, one co-expressing orexigenic AgRP and NPY and the other consisting of pro-opiomelanocortin (POMC) neurons in the ARC. The latter expresses the POMC gene, the precursor polypeptide encoded by which is cleaved into various neuropeptides, such as α-melanocyte-stimulating hormone (α-MSH), which exerts anorexigenic effects [10,37,38]. Peripheral signals—such as leptin, insulin, ghrelin, glucose, and fatty acids—modulate neuropeptide secretion by these neuronal populations, which influences food intake and energy balance, to maintain energy homeostasis [10,31].

Signals from the periphery to the central nervous system thus include gastrointestinal and endocrine signals [30]. Gastrointestinal hormones such as peptide YY (PYY), glucagon-like peptide-1 (GLP-1), cholecystokinin, and ghrelin are secreted in response to changes in macronutrient composition [39], reach the central nervous system via the bloodstream, and generate short-term signals to initiate or terminate food intake [40]. Leptin, secreted by adipose tissue and acting as a satiety signal, is also an important hormone for energy balance. It functions as an afferent signal in a negative feedback loop that regulates food intake and metabolism to maintain the homeostatic control of fat mass [41]. However, when it is absent, reduced, or inadequately perceived, it markedly increases the sensation of hunger [40].

In the transmission of peripheral signals, visceral information is carried by the vagus nerve from the gut to the brainstem for processing and is then relayed to the hypothalamus. Vagal afferents directly transmit information about the type and amount of ingested food, while vagal efferents, together with the sympathetic nervous system and hormonal mechanisms, regulate nutrient absorption, digestion, storage, and mobilization [42,43]. This signaling is crucial to maintaining energy homeostasis and regulating food intake. Any disruption in this signaling may lead to excessive eating and the development of obesity [43].

### 2.2. Non-Homeostatic Control of Food Intake

Homeostatic and non-homeostatic processes in the brain are closely integrated and interrelated [44]. While a negative energy balance primarily triggers the physiological need to eat through the hypothalamus and brainstem, the desire to consume palatable foods can stimulate food intake independently of the energy status [45]. Such non-homeostatic food intake, or “hedonic” eating, is driven by cognitive, emotional, and rewarding factors and occurs independently of metabolic feedback [44]. The initiation of eating is generally associated with non-homeostatic mechanisms, whereas meal size and termination are regulated by homeostatic processes controlled by gut signals linked to food intake [45].

The brain’s reward system comprises the components “liking” and “wanting,” which are regulated by complex and interconnected neural pathways, and is modulated by various factors, including emotional states, physiological conditions, social norms, and repeated exposure to food [46]. Neural reward circuits provide the anatomical and functional basis of hedonic regulation and respond to taste, smell, or visual cues associated with food. The consumption of a food linked to a pleasant sensation promotes a behavioral response that develops into a learned preference. Conversely, exposure to food associated with an unpleasant sensation leads to learned avoidance [47].

The regulation of hedonic responses to food is mediated by various neurotransmitters, including endocannabinoids, opioids, and monoamines such as dopamine and serotonin [47]. These neurotransmitters establish reciprocal connections among reward-related brain structures and act as second messengers to convey peripheral metabolic signals to the central nervous system [40]. Dopamine in the dorsal striatum generates survival-driven motivation to eat via D2 receptors, whereas nucleus accumbens is responsible for pleasure-driven motivation to eat. Serotonin plays a role in reducing the intake of palatable foods and is particularly important in the pathogenesis of eating disorders such as anorexia nervosa [47]. “Wanting” markedly influences eating behavior through dopaminergic pathways via cognitive and conditioned learning processes, whereas “liking” is regulated by endocannabinoid and opioid pathways [40]. The mesocorticolimbic system plays a central role in the reward mechanism, with the hippocampus, amygdala, prefrontal cortex, and midbrain being other key structures involved in this process. The lateral hypothalamus serves as an integrative center for homeostatic and hedonic systems and plays a key role in regulating hedonic eating through its connections with the ventral tegmental area and nucleus accumbens [47].

## 3. The Ghrelin System and Its Role in Eating Behavior

### 3.1. The Discovery and Structure of Ghrelin

Ghrelin was discovered in 1999 as an endogenous ligand for growth hormone secretagogue receptor (GHS-R1a) [48]. It is a 28-amino acid peptide primarily secreted by X/A-like neuroendocrine cells in the oxyntic glands of the gastric fundus [49,50]. Ghrelin mRNA has also been detected in the kidney [51], pancreas [52], lungs, intestine, and pituitary gland [53]. Its structure is highly conserved across species, with rat and human ghrelin peptides differing by only two amino acids. Ghrelin is produced by the proteolytic cleavage of a 117-amino acid precursor protein called preproghrelin. During this process, a medium-chain fatty acid is attached to the serine-3 residue. This acylation, catalyzed by the enzyme ghrelin O-acyltransferase (GOAT), leads to the formation of acyl ghrelin, which is necessary for the biological actions of ghrelin. The receptor mediating the effects of des-acyl ghrelin has not yet been clearly identified [50].

**Figure 1:**
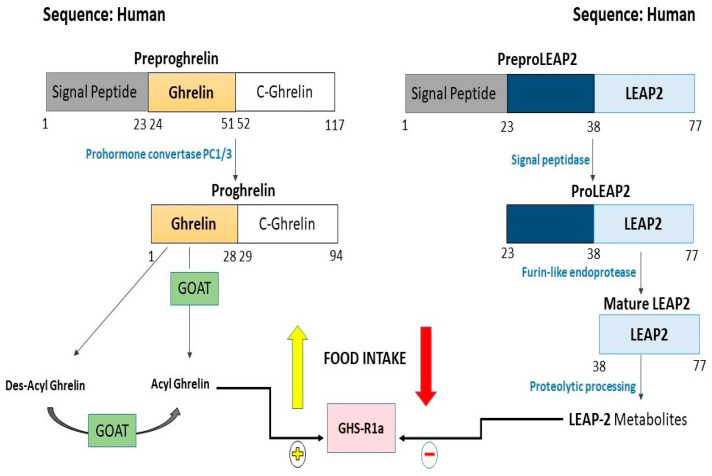
Human ghrelin and LEAP2: processing pathways and impact on food intake. Abbreviations: GOAT: ghrelin O-acyltransferase; GHS-R1a: growth hormone secretagogue receptor-1a. This figure was created by the authors using PowerPoint, inspired by pathway illustrations in References [28,50,54].

The human ghrelin gene consists of four exons. The main mRNA transcript of the ghrelin gene (transcript A) is translated into preproghrelin, the 117-amino acid precursor. This is converted into proghrelin by the prohormone convertase PC1/3 enzyme. The signal peptide sequence of preproghrelin (1–23) is cleaved to produce proghrelin (1–94). A series of post-translational steps, including protease cleavage and the acyl modification of the ghrelin precursor peptide, ultimately lead to the production of mature ghrelin peptides (acyl and des-acyl ghrelin) or other ghrelin gene-derived peptides (Figure 1) [28].

#### 3.1.1. Acyl Ghrelin

Kanamoto et al. (2001) first demonstrated that human medullary thyroid carcinoma cells can produce des-acyl ghrelin (DAG) and enzymatically convert it into acyl ghrelin (AG) [55]. Then, in 2008, Gutierrez et al. [56] identified ghrelin O-acyltransferase (GOAT) as the key enzyme catalyzing ghrelin acylation in a cell culture model [56]. GOAT-catalyzed acylation is necessary for ghrelin to initiate signaling through its receptor, growth hormone secretagogue receptor 1a (GHS-R1a), and exert its endocrine functions [28,57]. Acyl modifications are generally observed in receptors and integral membrane proteins; ghrelin represents the first example of acylation in a secreted protein [28].

In healthy individuals, ghrelin circulates in plasma at an average concentration of 117 ± 37 fmol/mL. The AG form accounts for only about 10% of total plasma ghrelin [58], whereas the remaining 80–90% is DAG [48].

#### 3.1.2. Des-Acyl Ghrelin

The effects and metabolism of des-acyl ghrelin in the body are still not fully understood. One view suggests that DAG exerts important physiological actions through an unknown non-GHS-R1a receptor [57,59,60]. A second view proposes that its effects require acylation by GOAT and the subsequent activation of GHS-R1a [61]. Studies supporting the latter have suggested that there may be a novel branch of the ghrelin signaling pathway [61,62,63] involving the re-acylation of ghrelin at the cellular site where signaling occurs [63]. Murtuza et al. [62] reported the presence of GOAT in the hippocampus and its ability to acylate DAG [62]. The local re-acylation of des-acyl ghrelin adds a new dimension to ghrelin signaling, as it indicates that cells and tissues expressing both GOAT and GHS-R1a can integrally sense the total circulating ghrelin concentration (acyl ghrelin + des-acyl ghrelin) [63].

### 3.2. Ghrelin and Appetite Regulation: Homeostatic and Hedonic Pathways

Ghrelin exerts orexigenic effects via three main pathways [12,64]:

- Once synthesized in the stomach, it travels through the bloodstream to the arcuate nucleus (ARC) and other parts of the brain, crossing the blood–brain barrier via active transport and influencing appetite.
- Peripherally synthesized ghrelin stimulates vagal afferent nerve endings and is transmitted to the hypothalamus via the nucleus solitarius.
- It is synthesized locally in the hypothalamus and directly stimulates Neuropeptide Y/Agouti-Related Peptide (NPY/AGRP) and other cells in the ARC [12,64].

In the fasting state, ghrelin promotes feeding by activating AgRP/NPY neurons in the paraventricular nucleus (PVN) and lateral hypothalamus (LH) [36]. In satiety, ghrelin release by the stomach decreases, and so does AgRP/NPY activity; POMC neurons are activated, and α-MSH (melanocyte-stimulating hormone) is secreted to activate MC4R receptors, which leads to the suppression of appetite [36].

The results on the effects of DAG on food intake are contradictory. In one study, DAG was reported to decrease food intake [65], while in another study, it was reported to suppress the appetite-increasing effect of peripherally administered ghrelin, but it did not have an appetite-increasing or -decreasing effect on its own [66].

Ghrelin also enhances the rewarding properties of certain foods, particularly those rich in sugar and fat, thereby promoting hedonic eating behavior. This effect is mediated by the dopaminergic system, as ghrelin stimulates dopamine release from VTA neurons and increases their action potential frequency [50].

### 3.3. Ghrelin and Metabolism

Some of the physiological roles of AG are the regulation of food intake, the control of GH secretion from the pituitary gland, the suppression of insulin secretion in the pancreas [67], the induction of adiposity, and the acceleration of gastrointestinal motility [68]. In recent years, it has been reported that DAG may have a regulatory effect on glucose [69,70] and lipid metabolism [70,71].

#### 3.3.1. Effects on Glucose Metabolism

Hedback et al. [26] investigated the effect of acyl ghrelin on pancreatic insulin secretion by administering acyl ghrelin (1 pmol/kg/min) or placebo infusions during a mixed-meal test and an ad libitum meal test. The study timepoints were before sleeve gastrectomy (SG) and 3 months after surgery. Additionally, at the latter timepoint, two different doses of acyl ghrelin infusion (0.25 pmol/kg/min and 10 pmol/kg/min) were administered on different experimental days. The acyl ghrelin infusions were reported to increase postprandial glucose levels and suppress insulin production both before and after surgery; specifically, they inhibited both basal and postprandial insulin secretion rates, resulting in decreased β-cell function measurements, but had no effect on insulin sensitivity. It was also noted that ad libitum food intake during the infusion did not differ compared with the placebo group [26]. In another study, Tong et al. [72] reported that low-dose (0.26 μg/kg/h) and high-dose (2.0 μg/kg/h) AG infusions in healthy individuals reduced insulin sensitivity, impaired β-cell function, and adversely affected glucose tolerance during the meal tolerance test compared with the control group. Zang et al. [69] reported that DAG levels were negatively associated with excess body fat mass and insulin resistance, whereas AG levels were associated with elevated blood glucose levels in patients with type 2 diabetes mellitus [69]. In a study by Özcan et al. [73], it was reported that DAG administration increased postprandial glucose levels without an increase in insulin levels in obese diabetic individuals, suggesting a potential beneficial effect on insulin sensitivity [73].

#### 3.3.2. Effects on Lipid Metabolism

Considering the lipogenic effect of AG on visceral adipocytes, elevated circulating AG concentrations in obese individuals may contribute to excessive fat accumulation in obesity [71]. In a study by Elibol et al. [70] in rats, the effects of AG and DAG on hepatic steatosis and glucose metabolism were investigated. The authors administered subcutaneous injections with placebo, AG (200 ng/kg), DAG (200 ng/kg), AG/DAG:1 (100/100 ng/kg), AG/DAG:3 (150/50 ng/kg), and AG/DAG:1/3 (50/150 ng/kg). AG was found to increase hepatic steatosis, whereas DAG reduced this effect. The highest PPAR-γ mRNA gene expression was observed in the AG/DAG 1:3 group, and DAG was found to be more effective in regulating blood glucose levels [70]. In the study, it was reported that the AG/DAG ratio should also be evaluated, instead of measuring only AG or DAG levels, and that changes in this ratio may also alter metabolic effects [70]. In a study by Elbaz and Gershon [74] in mice, DAG was reported to promote the utilization of fatty acids as metabolic fuel in muscle cells under conditions of food deprivation and low glucose levels.

#### 3.3.3. Effects on Gastrointestinal Motility

Ghrelin has been reported to be a potent stimulant of gastric emptying and gastrointestinal motility [75]. AG has been shown to increase motility in the antrum and duodenum [76]. These effects make the ghrelin system a therapeutic target to improve gastrointestinal system diseases or symptoms [77].

## 4. LEAP2: A Novel Antagonist in the Ghrelin System

LEAP2 was first identified in human blood ultrafiltrate by Krause in 2003 [78]. Subsequently, its presence was also detected in chickens (Gallus gallus) [79] and rainbow trout (Oncorhynchus mykiss) [80]. Krause defined LEAP2 as “Liver Expressed Antimicrobial Peptide 2” [78]. Although this term continues to be used in some recent publications, the correct term approved by the HUGO Gene Nomenclature Committee (HGNC) is “Liver Enriched Antimicrobial Peptide 2”. The abbreviation LEAP2 is also sometimes referred to as LEAP-2, and both forms are considered correct [81].

LEAP2 is known to play a role in the innate immune system, as it disrupts bacterial membrane integrity. In 2018, it was identified as the first endogenous antagonist of the ghrelin receptor (GHS-R1a). This information has emerged as the only known mechanism controlling the regulation of ghrelin secretion. LEAP2, with its 40-amino acid bicyclic structure containing two disulfide bridges, resembles many peptide hormones, so it was initially considered a potential hormone [16]. However, it was later defined in rodents as an endogenous circulating hormone that antagonizes the effects of ghrelin and exerts inverse effects on GHS-R structural activity [17]. Its biological functions have not yet been fully elucidated [16,17].

The human LEAP2 precursor protein weighs approximately 8.814 kDa and is synthesized as a 77-amino acid prepropeptide. After post-translational modification, the largest mature form of LEAP2 contains residues 38–77 and has a molecular weight of 4.585 kDa [78]. The first 22 amino acids (residues 1–22) function as a signal peptide, while residues 23–37 are truncated and removed during maturation. PreproLEAP2 is processed by signal peptidase and furin-like endoprotease into proLEAP2 and then into the 40-residue mature LEAP-2. The latter, consisting of residues 38–77, is subsequently degraded into small metabolites through proteolytic processing (Figure 1) [54]. Disulfide bonds are formed between residues 54 and 65 and between residues 60 and 70 to ensure the stability of the structure. Other forms of LEAP2 can be found circulating in the blood, such as 39–77, 43–77, 48–77, 48–77, 48–73, 48–75, 46–76, and 48–76, indicating structural flexibility. LEAP2 also has structural features characteristic of antimicrobial peptides, including a large number of positively charged amino acids that facilitate its interaction with Gram-positive bacteria [78,80].

LEAP2, produced in the liver and small intestine, prevents ghrelin binding to GHS-R, completely inhibiting the activation of the receptor. It has indeed been reported to block the main effects of ghrelin–receptor binding, including food intake, growth hormone (GH) release, and the maintenance of serum glucose levels in chronic energy restriction [16]. Further, LEAP2 has also been reported to inhibit the effects of ghrelin by disrupting additional mechanisms, such as its production and secretion [16].

It has been reported that ghrelin levels are lower in obese individuals compared with normal individuals [14], as also found for serum AG and DAG levels in a recent meta-analysis study conducted by Wang et al. [82]. In another study, it was reported that in obesity and obesity-related T2D, circulating acylated ghrelin concentrations increased, while des-acyl ghrelin levels decreased, and Body Mass Index (BMI), waist circumference, and insulin were positively correlated with acyl ghrelin levels [71]. Similarly, plasma LEAP-2 levels are affected by both long-term metabolic status (e.g., body mass and adiposity) and short-term meal-related changes. Many of these changes have been reported to have a trend opposite to that of ghrelin. It has been shown in both mice and humans that plasma LEAP-2 levels are approximately 20-fold higher than ghrelin levels, especially in the case of ad libitum feeding [14]. Obese mice have been reported to have lower plasma ghrelin and higher LEAP2 levels, as well as impaired transport of ghrelin across the blood–brain barrier, compared with normal-weight mice. This supports the hypothesis that resistance to ghrelin occurs in the hypothalamic system in obesity [15].

Although circulating ghrelin levels are reduced in obesity, peripheral ghrelin administration has been reported not to induce food intake in obese mice. There is limited information on ghrelin resistance associated with LEAP2 [83]. Obesity has been associated with increased LEAP2 levels and low ghrelin levels [84]. Lucie et al. [83] reported that both acyl and total plasma ghrelin levels decreased and liver LEAP2 mRNA expression increased in obese mice fed a high-fat diet. It was reported that liver LEAP2 mRNA expression and AG plasma levels normalized with the transition from a high-fat diet to a standard diet, while the total ghrelin levels did not change [83]. Gradel et al. [85] reported that a high-fat diet increased plasma LEAP2 levels compared with a standard diet in mice. It has been reported that LEAP2 is upregulated in the state of positive energy balance in obesity and diabetes; this may be a counter-regulatory mechanism to prevent a further increase in energy stores [85]. LEAP2 has been reported to be a promising therapeutic target for obesity and other metabolic diseases, with the view that further increasing the LEAP2–ghrelin molar ratio may help restrict food intake [14,84].

Ghrelin and LEAP2 have been shown to play opposite roles in food intake modulation. However, in vivo studies on LEAP2 are still very limited, and some fundamental questions about the interaction between the two molecules remain unanswered [86]. In a study by Wald et al. [87] investigating whether LEAP2 inhibits ghrelin-induced feeding, it was reported that the administration of LEAP2 decreased food intake and body weight in individuals on a normal diet and also inhibited the feeding-stimulating effect of ghrelin. In a similar study by Lugilde et al. [86], centrally administered exogenous LEAP2 produced a significant anorexigenic (appetite-suppressing) effect in three different rodent animal models, including lean, obese, and aged models, and significantly suppressed ghrelin-induced food intake. In a study by Chu et al. [88] examining the effects of LEAP2 on the arcuate nucleus of the hypothalamus (ARC), the overexpression of LEAP2 in the ARC with adeno-associated virus (AAV) suppressed both central and peripheral ghrelin effects in mice fed a standard diet; further, chemogenetic analyses showed that the inhibition of POMC neurons in the ARC abolished the anorexigenic effect exerted by LEAP2 [88]. These results suggest that the central administration of LEAP2 produces appetite-suppressing and body weight-reducing effects, which most likely require the activation of POMC neurons in the ARC [88]. Hagemann et al. [89] reported that LEAP2 infusion reduced postprandial plasma glucose and growth hormone concentrations and decreased food intake during the ad libitum meal test. In a study on LEAP2 and hedonic systems, it was reported that hedonic eating behavior was suppressed, dopamine levels decreased, and desire for high-energy foods decreased after LEAP2 administration in mice [90].

## 5. Changes in Acyl Ghrelin, Des-Acyl Ghrelin, and LEAP2 After Bariatric Surgery

Bariatric surgery is a gastrointestinal surgical intervention primarily performed for weight loss in obese individuals; even if patients do not significantly lose weight, they still experience improvement in metabolic diseases [91]. Bariatric surgery is regarded as the most effective weight loss method because it restricts gastrointestinal tract volume and/or induces beneficial metabolic effects [20,21]. Weight loss due to bariatric surgery occurs through multiple mechanisms beyond mechanical restriction or malabsorption, as the gut–brain axis is also targeted through the release of gut hormones, nutrient sensing, and bile acid signaling. In this intervention, a number of factors are known to act together to synergistically alter feeding behavior and prevent weight gain [92], with the effects varying according to the type of surgery. There are different bariatric surgery procedures, i.e., gastric bypass, sleeve gastrectomy, gastric banding, and biliopancreatic diversion, divided into three subgroups: volume-restricting procedures, absorption-impairing procedures, and surgeries with both effects. Gastric banding and sleeve gastrectomy are restrictive, biliopancreatic diversion is malabsorptive, and gastric bypass is both restrictive and malabsorptive [93]. According to the results of IFSO, from 2008 to 2016, sleeve gastrectomy (SG) was the most common bariatric procedure performed worldwide, followed by Roux-en-Y gastric bypass [94]. According to the latest data from IFSO, there was a significant decrease in the number of metabolic and bariatric surgery procedures worldwide in 2020. In 2021, although an increasing trend was observed, a return to pre-pandemic values was not observed. However, adjustable gastric band (AGB) applications continued to decrease worldwide, and it has been reported that SG continues to be the most frequently performed surgical procedure [95].

Sleeve gastrectomy is an operation performed to create a tubular stomach by removing the large curvature of the stomach. Numerous studies have shown that sleeve gastrectomy, due to the removal of the gastric fundus, leads to a significant decrease in plasma ghrelin levels, which has been associated with decreased appetite and consequent weight loss [96,97,98], as well as an increase in satiety hormones such as PYY and GLP-1 [99,100]. It has been reported that gastric emptying and small intestinal transit are accelerated after SG, which may contribute to changes in nutrient absorption and metabolism [101,102]. SG has also been reported to reduce the preference for high-fat, high-sugar, and high-energy foods, which suggests reduced hedonic drive to consume such foods [103,104].

In RYGB surgery, a small gastric pouch is created in the upper part of the stomach, significantly reducing the total stomach volume (restrictive effect). This pouch is separated from the rest of the stomach, which remains in place but is no longer in contact with food. Further, a segment of the small intestine (specifically the jejunum) is cut, and one of its ends (the Roux limb) is connected to this gastric pouch, while the other (biliopancreatic limb) is reconnected to a lower part of the small intestine. In this way, digestive fluids from the stomach, liver, and pancreas mix with food further down the intestine, where digestion and absorption take place (malabsorptive effect) [105]. Appetite after this surgical intervention on the gastrointestinal system has been reported to decrease, a change that is partly attributed to alterations in gastrointestinal hormone secretion [106,107]. Fasting total ghrelin levels after RYGB have been reported to decrease in the short term (≤3 months) but to increase in the long term (>3 months) [108]. The fact that the stomach is not removed in standard RYGB is thought to contribute to the more heterogeneous findings regarding ghrelin levels and clinical outcomes compared with SG. However, one study reported that performing fundus resection in RYGB did not produce significant differences in clinical outcomes, such as an enhancement in weight loss, a reduction in appetite, or an increase in satiety [109]. In contrast, another study reported that following RYGB, fasting ghrelin levels decreased after 3 months but rose above baseline levels after 12 months; however, after RYGB with fundus resection, ghrelin levels remained significantly and persistently low, and postprandial GLP-1, PYY, and insulin responses increased to a greater extent [110].

Table 1 presents the changes in acyl ghrelin (AG), des-acyl ghrelin (DAG), and LEAP2 levels, rather than total ghrelin levels, after bariatric surgery. Most studies have reported a significant reduction in fasting AG [14,23,26,111] and DAG [112,113] levels following SG. However, there is heterogeneity in the results due to differences in sample types, study populations, and analysis methods. The accurate assessment of gut hormone levels requires the standardization of sampling, adherence to the study protocol, and rigorous sampling and handling for unstable hormones such as acyl ghrelin [114]. Attention to these methodological aspects is critical to reducing the discrepancies in the literature and fully leverage the potential of gut hormones in the treatment of obesity.

In the examined studies, although most of the gastric fundus was removed, fasting plasma acyl ghrelin levels were reported to decrease by approximately 25% 6 weeks after sleeve gastrectomy (SG) [23], by 80% after 3 months [26], and by 40% after 6 months [113]. In the latter, in the same patient group, these levels increased slightly after 12 months compared with 6 months but remained approximately 20% lower than preoperative levels [113]. One possible explanation for this is that although the majority of ghrelin-producing cells are located in the fundus, the source of circulating acyl ghrelin may originate from the duodenum. It is also possible that a compensatory increase in duodenal ghrelin production occurs following the removal of the fundus to maintain hormonal balance [23]. Therefore, the heterogeneity of the results in the studies may be due to the differences in the study periods.

Changes in the levels of the ghrelin isoforms appear to be more pronounced following sleeve gastrectomy (SG) than after gastric bypass procedures (Table 1). This difference may be attributed to the anatomical resection of the fundus in SG, which is the primary site of ghrelin secretion, leading to a greater impact on circulating ghrelin levels. Structural and physiological differences between surgical techniques represent a key source of heterogeneity. Therefore, the type of surgical procedure performed should be taken into account when comparing findings across studies in the literature.

While an increase in LEAP2 was observed after meals in obese individuals preoperatively, this increase was not seen after SG and RYGB [14]; on the contrary, a significant decrease in postprandial plasma LEAP2 levels was observed [14]. It has been reported that the decrease in plasma LEAP2 levels after bariatric surgery may be related to weight loss rather than the intervention itself [81]. However, the number of studies investigating LEAP2 levels after bariatric surgery remains limited (Table 1). Further research is needed to assess changes in LEAP2 levels after SG, particularly in relation to ghrelin, and their impact on food intake. Considering that the LEAP2–ghrelin interaction is receptor-mediated, studies focusing on the receptor-binding acyl ghrelin form rather than total ghrelin levels are important for better defining this relationship.

## 6. Limitations

Although the association of bariatric surgery with changes in the levels of the ghrelin isoforms has been extensively studied, research on LEAP2 is limited. Therefore, some important limitations need to be considered. The heterogeneity in the study results regarding changes in ghrelin isoforms is largely due to differences in methods, patient groups (BMI, gender, presence of insulin resistance, etc.), surgical techniques, and follow-up periods. Furthermore, the limited number of studies investigating LEAP2 levels after bariatric surgery makes it difficult to draw clear inferences regarding its role in appetite regulation and metabolic outcomes. Most of the existing studies have focused on total or fasting ghrelin levels, and the receptor-binding form of acyl ghrelin, which may be more relevant in understanding the interaction of ghrelin and LEAP2, has not been adequately investigated.

## 7. Research Gaps and Future Perspectives

The ghrelin–LEAP2 relationship is a new area as well as a hotspot of obesity and bariatric surgery research. However, the current literature lacks sufficient comprehensive and comparative studies investigating the ghrelin isoforms and LEAP2 levels. Future research employing standardized methodologies, extended follow-up periods, and larger participant groups is warranted to better elucidate the ghrelin–LEAP2 interplay and its clinical implications.

Future research should focus on the following key areas:

- Interactions at the Receptor Level: Molecular interactions between LEAP2 and ghrelin—particularly those involving acylated ghrelin—at the receptor level should be further investigated. This may provide a more accurate understanding of the regulatory role of this axis in appetite and metabolism.
- Diet-Related LEAP2 Regulation: The effects of various dietary components and meal patterns on LEAP2 expression should be investigated, especially in individuals with obesity or metabolic syndrome.
- Pharmacological Potential of LEAP2: The antagonistic effect of LEAP2 on the ghrelin receptor makes it a promising therapeutic target for appetite and weight management. However, further clarification of its physiological effects is needed to support this potential. Studies examining its efficacy, safety and pharmacokinetic properties are also needed.
- Relationship with Hedonic Hunger: Future studies should also address the role of LEAP2 in hedonic hunger and reward-driven eating, which may shed light on clinically important issues such as overeating behaviors and dietary failures in the obese.
- Long- and Short-Term Effects of Bariatric Surgery: Further studies examining the effects of different types of bariatric surgery on acyl ghrelin and LEAP2 levels are needed. In particular, there remains a lack of data on the short- and long-term effects of these hormones on metabolic outcomes and appetite.
- Clinical Measurement Challenges: The measurement of LEAP2, acyl ghrelin, and des-acyl ghrelin levels in clinical settings is not yet widely applicable. Currently, these biomarkers are assessed under research conditions that require high-sensitivity analytical techniques and stringent sampling protocols. Therefore, there is a need to develop simpler, faster, cost-effective, and clinically feasible measurement methods to enable their use in routine clinical practice.

Advances in these areas will contribute to a better understanding of the mechanisms of action and allow for the development of new therapeutic targets in the treatment of obesity.

## 8. Conclusions

Ghrelin, an orexigenic hormone, plays an important role in appetite metabolism, including homeostatic and hedonic processes. In obesity, altered neural and hormonal responses to food also affect ghrelin metabolism. Ghrelin levels are lower in obese individuals than in normal-weight individuals, which leads to an inhibition of some of its metabolic effects. Similarly, plasma LEAP2 levels are influenced by both long-term metabolic status (e.g., body mass and adiposity) and short-term meal-related changes. Most of these changes have been reported to show a tendency opposite to that of ghrelin. After bariatric surgery, performed for rapid weight loss and metabolic improvement in the treatment of obesity, changes in ghrelin levels have been observed. Most studies have reported a significant reduction in fasting acyl ghrelin and des-acyl ghrelin levels following sleeve gastrectomy, with these changes appearing to be more pronounced in sleeve gastrectomy groups compared to gastric bypass groups. On the other hand, the number of in vivo studies evaluating LEAP2 levels after bariatric surgery is quite limited. Current studies have reported that LEAP2 may exert suppressive effects on ghrelin-induced food intake. However, many issues, such as the efficacy of LEAP2 as a pharmacological target in the treatment of obesity, its possible role in hedonic eating behaviors, and the changes in its levels depending on different types of bariatric surgery, are still poorly understood.

## Figures and Tables

**Table 1:** Levels of LEAP2, acyl ghrelin, and des-acyl ghrelin after bariatric surgery.

Hormone	Study/ Sample Type	Type of Surgery/Sample Size (*n*)	Follow-Up Period	Outcome	Ref.
**LEAP-2**					
	Retrospective observational/obese individuals	**VSG**/*n* = 39	12 mo	No change was observed in fasting serum LEAP2 levels.	[115]
	Experimental/C57BL/6J mice	**VSG**/*n* = 6 and 6 control	-	LEAP2 mRNA expression was almost undetectable in the stomach under normal physiological conditions, but it was reported to increase markedly after VSG surgery, while it decreased in the duodenum.	[16]
	Observational cohort/obese individuals	**RYGB**/*n* = 14	3 mo 2 y	Fasting plasma LEAP2 levels were reported to be significantly lower 2 years after RYGB (*n* = 8), but no reduction was observed 3 months after surgery (*n* = 14). Postprandial plasma LEAP2 levels were reported to have significantly decreased 3 months after RYGB (*n* = 11).	[14]
		**VSG**/*n* = 7	12 and 18 mo	A significant reduction in postprandial LEAP2 levels was reported 12–18 months after surgery.	
**Acyl Ghrelin**					
	Observational cohort/obese individuals	**VSG**/*n* = 7	12 and 18 mo	Fasting and postprandial plasma acyl ghrelin levels were reported to have significantly decreased approximately 12 to 18 months after surgery.	[14]
	Experimental/obese individuals	**SG**/*n* = 12	3 mo	Fasting plasma AG levels were reported to have significantly decreased.	[26]
	Prospective observational/obese individuals	**SG**/*n* = 8	6 wk, 3 mo	Fasting plasma AG levels were reported to have decreased 6 weeks and 3 months after surgery, with a more pronounced reduction compared with RYGB.	[23]
		**RYGB**/*n* = 10	6 wk, 3 mo	Fasting AG levels decreased insignificantly after 6 weeks but rose toward baseline values after 3 months. Postprandial acyl ghrelin levels at 30 min decreased both 6 and 3 months after intervention compared with pre-surgery levels.	
**Acyl Ghrelin**					
	Prospective observational/obese individuals	**SG**/*n* = 5, **RYGB**/*n* = 9, and **MGB**/*n* = 9	6 and 12 mo	Lower fasting AG levels in the SG group compared with the MGB group after 6 months were reported. No significant difference in AG levels between the SG and MGB groups after 12 months was reported.	[113]
	Experimental/Wistar rats	**SG**/*n* = 37	1 mo	Plasma fasting AG levels were reported to have remained unchanged after SG.	[112]
	Prospective observational/morbidly obese individuals	**SG**/*n* = 61	6 and 12 mo	Plasma fasting AG levels were reported to have significantly decreased 6 and 12 months after SG.	[111]
	Experimental/C57BL/6J mice	**RYGB**/*n* = 10	6 wk	Plasma fasting AG levels were reported to be significantly higher in the RYGB group compared with both ad libitum-fed obese mice (*n* = 10) and weight-matched, restricted-feeding obese mice (*n* = 10).	[116]
**Des-Acyl Ghrelin**					
	Experimental/Wistar rats	**SG**/*n* = 37	1 mo	A significant decrease in plasma fasting DAG levels after SG was reported.	[112]
	Prospective observational/obese individuals	**SG**/*n* = 5, **RYGB**/*n* = 9, and **MGB**/*n* = 9	6 and 12 mo	A significant reduction in fasting and postprandial DAG levels in the SG group after 6 and 12 months was reported. Lower fasting and postprandial DAG levels in the SG group compared with the RYGB group after 12 months were reported. Lower fasting DAG levels were found in the SG group compared with the MGB group after 6 months, but no significant difference in DAG levels between the SG and MGB groups after 12 months was reported.	[113]
	Prospective observational/morbidly obese individuals	**SG**/*n* = 61	6 and 12 mo	Plasma fasting DAG levels were reported to show a decreasing trend after 6 and 12 months, but this was not significant.	[111]
	Experimental/C57BL/6J mice	**RYGB**/*n* = 10	6 wk	Plasma fasting DAG levels were reported to be higher in RYGB-treated mice (*n* = 10) compared with ad libitum-fed obese mice (*n* = 10) and weight-matched, restricted-feeding obese mice (*n* = 10).	[116]

Abbreviations used: AG: acyl ghrelin; DAG: des-acyl ghrelin; LEAP2: liver-expressed antimicrobial peptide 2; MGB: mini gastric bypass; mo: month; Ref: reference; RYGB: Roux-en-Y gastric bypass; SG: sleeve gastrectomy; VSG: vertical sleeve gastrectomy; wk: week; y: year.

## Data Availability

No new data were created or analyzed in this study.
